# Effects of photocoagulation on ocular blood flow in patients with severe non-proliferative diabetic retinopathy

**DOI:** 10.1371/journal.pone.0174427

**Published:** 2017-03-29

**Authors:** Takeshi Iwase, Misato Kobayashi, Kentaro Yamamoto, Eimei Ra, Hiroko Terasaki

**Affiliations:** Department of Ophthalmology, Nagoya University Graduate School of Medicine, Nagoya, Showa-ku, Japan; Massachusetts Eye & Ear Infirmary, Harvard Medical School, UNITED STATES

## Abstract

**Purpose:**

To investigate ocular blood flow and correlations between ocular blood flow and variables in patients with severe non-proliferative diabetic retinopathy (S-NPDR) following panretinal photocoagulation (PRP).

**Methods:**

In this retrospective, cross-sectional study, the blood flow on the optic nerve head (ONH) and choroid was assessed with laser speckle flowgraphy (LSFG) using the mean blur rate (MBR) in 76 eyes of 76 patients with S-NPDR who underwent PRP, 39 eyes of 39 patients with S-NPDR who did not undergo PRP, and 71 eyes of 71 normal subjects. The correlation between MBR and variables, including visual acuity (VA) and choroidal area determined by binarization method, was analyzed.

**Results:**

The mean age was 62.9 ± 11.9 years in the S-NPDR with PRP eyes, 55.6 ± 11.4 years in the S-NPDR without PRP eyes, and 60.3 ± 11.1 years in the normal subject eyes. The ONH MBR in vessel and tissue areas and the choroidal MBR were significantly lower in the S-NDR with PRP group than in the other groups (*p* < 0.001, *p* < 0.001, and *p* < 0.001, respectively). The luminal and the stromal areas were significantly smaller in the S-NDR with PRP group than in the other groups (*p* < 0.001 and *p* < 0.001, respectively). LogMAR best corrected visual acuity (BCVA) exhibited significant negative correlation with the ONH MBR in vessel (r = −0.386, *p* < 0.001), tissue (*r* = −0.348, *p* < 0.001), and the choroid MBR (*r* = −0.339, *p* = 0.002) in the S-NDR with PRP group. Stepwise multiple regression analysis demonstrated that BCVA was a common independent factor associated with the ONH MBR in vessel, tissue, and the choroidal MBR in the S-NDR with PRP group.

**Conclusions:**

ONH and choroid MBR in addition to choroidal component, including the luminal area, were significantly lower in eyes of patients with S-NPDR after PRP compared with no PRP and normal subjects group. This could suggest that the significantly reduced ocular blood flow in PRP-treated S-NPDR eyes correlated with long-term decreased post-PRP luminal area and visual acuity.

## Introduction

Diabetic retinopathy is one of the leading causes of blindness in the industrialized world. Studies have demonstrated that panretinal photocoagulation (PRP) is a beneficial clinical treatment that reduces the incidence of blindness in patients with proliferative diabetic retinopathy (PDR).[1–3] A five-stage disease severity classification for diabetic retinopathy includes three stages of low risk, a fourth stage of severe non-PDR (S-NPDR), and a fifth stage of PDR. [4, 5] At least one of the following should be present in S-NPDR: a) "severe" haemorrhages and microaneurysms in all four quadrants of the fundus, b) venous beading, which is more marked in at least two quadrants, and c) intraretinal microvascular abnormalities, which are more severe in at least one quadrant. The Early Treatment Diabetic Retinopathy Study subsequently demonstrated PRP to be associated with maintenance of good long-term visual acuity in most patients with S-NPDR or PDR.[6]

It has been proposed that PRP improves the oxygenation of ischemic inner retinal layers by destroying some of the metabolically highly active photoreceptor cells, leading to a greater flow of oxygen from the choriocapillaris to the inner layers of the retina.[7] Animal studies have shown an increase in the oxygen delivered from the choriocapillaris to the inner retina after photocoagulation.[8]

Destruction of the retinal pigment epithelium (RPE) and outer retinal tissue by photocoagulation may influence the choroid’s circulation underneath. Many studies focusing on the effects of PRP on ocular circulation have reported that PRP reduces retinal blood flow in patients with diabetic retinopathy.[9–12] Measuring choroidal blood flow is particularly challenging because the choroidal vessels are three-dimentional and complex and are hidden from view by the RPE, which results in choroidal blood flow not be directly evaluated.

Choroidal blood flow represents the major supply of oxygen and nutrients to the choroid and outer retina. Accordingly, knowledge on choroidal blood flow is important for understanding pathological conditions.[13] Various techniques for measuring choroidal blood flow have been developed, including computerized pneumotonometry,[14] indocyanine green angiography,[15] and near-infrared Doppler flowmetry.[16] The clinical utility of available methods is hampered by the time-consuming nature of the procedures, which are therefore unsuitable for large-scale trials.

The choroid is mainly composed of vessels and stroma (extravascular tissue), lacking a well-organized structure. Therefore, it is important to understand the variations in the choroidal structure. However, it is difficult to differentiate the luminal area from the stromal area in the choroid *in vivo*. Recently, Sonoda et al reported the use of a binarization method involving optical coherence tomography (OCT) images, which can differentiate the choroidal luminal area from the stromal area and quantify these areas using a software, Image-J, with a high reproducibility.[17, 18]

Laser speckle flowgraphy (LSFG) (Softcare Co., Ltd., Fukutsu, Japan) is a non-invasive, real-time method used to measure the relative blood flow in the choroid and optic nerve head (ONH) for 4 s without the use of contrast agents.[19–21] LSFG can detect the speckle contrast pattern produced by the interference of illuminating laser light that is scattered by the movement of erythrocytes in the blood vessels and enables measurement of the relative blood flow in the vessels expressed as the mean blur rate (MBR).[19–21] LSFG values correlate well with the actual blood flow values determined using hydrogen gas clearance and microsphere methods,[22, 23] meaning that variables determined with LSFG would be comparable between individuals. Aizawa et al reported that the coefficient of variation for MBR was 4.7 for the choroid and 3.4 for the ONH.[24] Therefore, LSFG was considered to be suitable for measuring ONH and choroidal blood flow in large-scale trials.

To the best of our knowledge, no reports have so far included data on the long-term evaluation of retinal and choroid blood flow in individual patients with S-NPDR following PRP. Thus, the purpose of this study was to assess the effect of PRP on ocular blood flow and its potential correlation with variables such as the choroidal area determined by binarization in patients with S-NPDR.

## Methods

### Ethics statement

In this retrospective, cross-sectional single-center study, the procedures used were approved by the Ethics Committee of the Nagoya University Hospital (Nagoya, Japan). The study was performed at the Nagoya University Hospital, and the study conformed to the tenets of the Declaration of Helsinki. A written informed consent had been obtained from all of the patients for the PRP after an explanation of the procedures to be performed and possible complications. Permission was also obtained to use the data collected for future research.

### Subjects

Patients who had S-NPDR (type 2 DM) and had undergone PRP, did not have undergone PRP, and normal subjects without ocular and systemic diseases were recruited and included in the study at Nagoya University Hospital from April 2014 to April 2016. All subjects underwent a comprehensive ophthalmic examination including the measurement e.g., slit-lamp examination, fundus examination.

All subjects were examined with a view to identifying the presence of any ocular disease. Slit-lamp examination and indirect ophthalmoscopy were used to examine the anterior and posterior segments of the eye, respectively. Furthermore, normal subjects were screened for any medical condition that might influence the hemodynamics of the eye, such as diabetes, hypertension, arrhythmia, and vascular diseases. The exclusion criteria for groups included the presence of any macular abnormalities such as choroidal neovascularization or asymptomatic pigment epithelial detachment, a history of other ophthalmic disorders, incisional surgery in the experimental eye, topical anti-glaucoma treatment, systemic hormonal medications, or anti-VEGF therapy or steroid for diabetic macular edema at last 1 year before the measurements, or AL > 26.5 mm.[25]

The relative blood flow was determined by LSFG-NAVI instrument (Softcare, Fukuoka, Japan) as described below. Because alcohol [26] and caffeine [27] intake can influence IOP, all participants were asked to abstain from alcoholic and caffeinated beverages from the evening before and on the day of the study. Additionally, all participants were instructed to avoid food consumption from 2 h before each experiment. All examinations were performed in the sitting position and on the same day. Each subject rested for 10–15 min in a quiet room prior to the tests, and each experimental session was completed within 15 min. The best-corrected visual acuity (BCVA) was measured with a standard Japanese decimal VA chart and converted to the logarithm of the minimum angle of resolution (logMAR) units. Axial lengths were measured with a partial optical coherence interferometry (IOLMaster; Carl Zeiss Meditec, La Jolla, CA), and intraocular pressure (IOP) was measured with a handheld tonometer (Icare; Tiolat Oy, Helsinki, Finland). Systolic blood pressure (SBP) and diastolic blood pressure (DBP) were measured for the left brachial artery at the height of the heart in a sitting position with an automatic sphygmomanometer (CH-483C; Citizen, Tokyo, Japan). The mean arterial blood pressure (MAP) and mean ocular perfusion pressure (MOPP) were calculated as follows: MAP = DBP + 1/3(SBP − DBP) and MOPP = 2/3MAP − IOP, respectively.[28]

### PRP treatment

The criteria for performing PRP are that patients had non-perfusion retinal areas in three or more quadrants in fluorescein fundus angiography. PRP was performed through a wide-field contact lens using a slit-lamp adapted photocoagulator (Lumenis Novus Varia^®^; Lumenis Ltd., Yokneam, Israel) with yellow color according to the Early Treatment Diabetic Retinopathy Study protocol. [29] PRP for each eye was performed in 3–5 sessions with 2-week intervals between sessions. For each session, photocoagulation was performed with 200-mm spot sizes with pulse duration of 0.2 seconds. 400–600 spots were made for a total to 1,200–3,500 spots to obtain a complete PRP. The power of the laser was individually adjusted to produce yellowish-white coagulative spots and ranged between 100 and 200 mW.

### Laser speckle flowgraphy

LSFG-NAVI was used to determine the relative ocular blood flow. The principles of LSFG have been described in detail elsewhere.[30–32] Briefly, this instrument comprises a fundus camera equipped with an 830-nm diode laser and a charge-coupled camera (750 width × 360 height pixels). After switching on the laser, a speckle pattern appears because of the interference of the light scattered from the illuminated tissue. MBR is a measure of the relative blood flow and is determined by examining the pattern of the speckle contrast produced by the interference of the laser light that is scattered by the movement of the blood cells in the ocular blood vessels. MBR images are acquired at a rate of 30 frames/s over a 4-s period. The embedded analysis software synchronizes all MBR images with each cardiac cycle, and the averaged MBR of a heartbeat is displayed as a heartbeat map.

**Fig 1:**
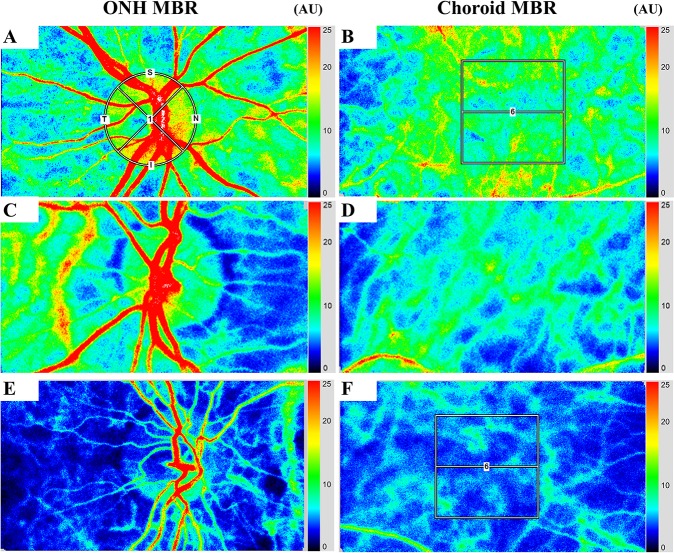
Representative composite color maps reflecting the mean blur rate (MBR) as measured by laser speckle flowgraphy (LSFG). Red color indicates a high MBR, and blue color indicates a low MBR. To measure the MBR of the optic nerve head (ONH) blood flow and choroidal blood, a circle was set around the ONH (left) and the center of a rectangle was set at the fovea (250 × 250 pixels, degree: 6.31° × 6.31°) (right). Eyes of i) normal subjects (A, B), ii) severe non-proliferative diabetic retinopathy (S-NPDR) patients without panretinal photocoagulation (PRP) (C, D), and iii) S-NPDR patients with PRP (E, F) are demonstrated. The blue color is dominant in the ONH and choroid in S-NPDR patient eyes with PRP (E, F).

To evaluate the changes in ONH and choroidal blood flow, a circle was set surrounding the ONH (Fig 1A), and a rectangle (250 × 250 pixels) was placed around the macula (Fig 1B). The software in the instrument was able to track the eye movements during the measurement period. LSFG was measured twice for each time point in all of the eyes. Average MBR values were calculated for each circle or rectangle using the LSFG Analyzer software (v.3.1.59).

### Measurement of Subfoveal choroidal thickness (SFCT)

Choroidal images were obtained by spectral-domain OCT (SD-OCT; Spectralis OCT, Heidelberg Engineering, Heidelberg, Germany). SD-OCT was placed close enough to the eye to obtain inverted images as previously described.[33] The subfoveal choroidal thickness (SFCT) was measured as the distance from the hyper-reflective RPE line to the choroid–sclera border with a caliper tool on SD-OCT by two experienced clinicians who were blinded to other study parameters.

### Differentiation of luminal and stromal areas

**Fig 2:**
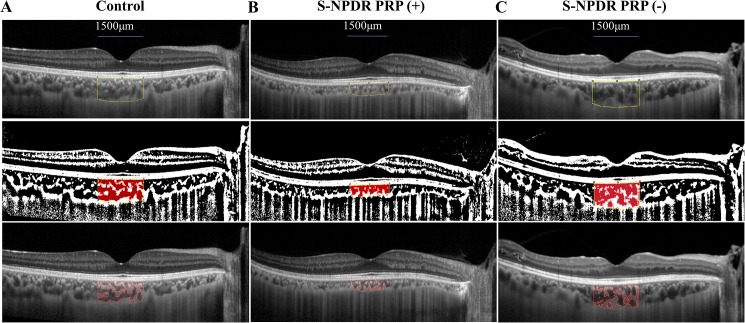
Representative binarization image of a choroidal area in an enhanced depth imaging (EDI) optical coherence tomography (OCT) image. The eyes with normal subject (A), severe non-proliferative diabetic retinopathy (S-NPDR) patients with panretinal photocoagulation (PRP) (B), and S-NPDR patients without PRP (C) are demonstrated. The area of interest of the choroid is demarcated (top). The EDI-OCT image was converted to a binary image using ImageJ software. The rectangle surrounded by the red line was excised, and the dark areas were traced by the modified Niblack method (middle). The binarized image and the margin of the traced area were merged, demonstrating that the traced area represented the luminal area, being consistent with the dark areas of the choroidal areas observed in the OCT image (bottom). The choroidal and luminal areas in the eyes of S-NPDR patients with PRP were smaller than those in the eyes of normal subjects or S-NPDR patients without PRP.

Binarization of the choroidal area in EDI-OCT images was performed by the modified Niblack’s method as previously reported.[17] EDI-OCT images were analyzed using the ImageJ software (ImageJ version 1.47, NIH, Bethesda, MD, USA). The examined area was 1,500 μm wide in the subfoveal choroid, extending vertically from the RPE to the chorioscleral border (Fig 2). This choroidal area was selected using the ImageJ ROI Manager. Next, the image was converted into 8 bits. The vitreous cavity in front of the macular area was selected by the Oval Selection Tool on the ImageJ tool bar, and the maximum reflectivity of these areas was determined. The maximum brightness was set at the minimum value to minimize noise in the OCT image. After adjusting by the Niblack Auto Local Threshold, the luminal area was determined using the threshold tool. The light pixels were defined as the stromal areas, and the dark pixels were defined as the luminal areas. After adding the data of the distance of each pixel, the luminal and stromal areas were automatically calculated. Two clinicians blinded to the other findings measured the area.

### Statistical analyses

The value of each parameter was presented as the mean ± standard deviation. For comparisons of categorical data, the chi-square test was used, whereas quantitative data were compared using Kruskal–Wallis tests. Analysis of variance with post-hoc Bonferroni correction was used to evaluate data pertaining to blood flow parameters and choroidal areas. Spearman’s rank correlation coefficient tests were used to determine the correlation coefficients between the variables. Multiple stepwise regression analysis was used to determine the association between blood flow parameters and other variables. All statistical analyses were performed using IBM SPSS Statistics for Windows, v.23 (IBM Corp., Armonk, NY). The significance level was set at a probability (*p*) value < 0.05.

## Results

### Demographics of subjects

Seventy-six eyes of 76 patients with S-NPDR who underwent PRP, 39 eyes of 39 patients who did not undergo PRP, and 71 eyes of 71 normal subjects were enrolled in this study. Demographic data on all subjects are shown in Table 1. No significant differences were observed in terms of gender, IOP, axial length, DBP, MOPP, or HR among the groups, while there were significant differences in age (*p* < 0.001), BCVA (*p* < 0.001), SFCT (*p* < 0.001), and SBP (*p* = 0.005) among the groups.

**Table 1:** Clinical characteristics of subjects.

Characteristics	S-NPDR with PRP (n = 76)	S-NPDR without PRP (n = 39)	Normal subjects (n = 71)	*p*—value
Age (years)	62.9 ±11.9	55.6 ±11.4	60.3 ± 11.1	< 0.001
Gender (male: female)	46: 30	24: 15	32: 39	0.111
HbA1c (%)	7.3 ± 1.3	8.8 ± 2.1	-	< 0.001
Duration of diabetes (years)	19.4 ± 9.4	11.8 ± 7.8	-	< 0.001
Insulin: oral hypoglycemic agents	38: 38	20: 19	-	0.897
Duration after photocoagulation (year)	8.4 ± 5.6	-	-	-
Best corrected visual acuity (Log MAR)	0.29 ± 0.29	0.09 ± 0.11	-0.00 ± 0.05	< 0.001
Intraocular pressure (mmHg)	14.1 ± 2.8	15.1 ± 2.8	13.8 ± 2.7	0.685
Axial length (mm)	23.80 ± 1.18	23.84 ± 1.01	24.17 ± 1.38	0.163
Subfoveal choroidal thickness (μm)	222.3 ± 85.9	292.3 ± 48.3	256.9 ± 70.9	< 0.001
Systolic blood pressure (mmHg)	136.3 ± 24.4	131.4 ± 29.7	127.2 ± 13.7	0.048
Diastolic blood pressure (mmHg)	78.6 ± 15.4	77.6 ± 16.1	78.2 ± 9.4	0.925
Ocular perfusion pressure (mmHg)	51.2 ± 11.9	48.6 ± 13.2	49.2 ± 7.4	0.379
Heart rate (bpm)	76.9 ± 11.9	77.3 ± 9.5	73.5 ± 10.6	0.096

S-NPDR = severe non-proliferative diabetic retinopathy.

### Comparison of ocular blood flow between eyes with diabetic retinopathy and normal subjects

**Fig 3:**
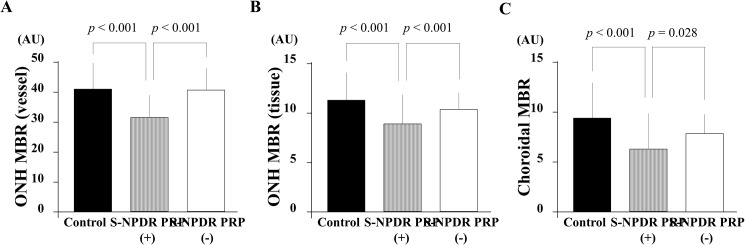
Differences between the eyes of normal subjects and those of patients with severe non-proliferative diabetic retinopathy (S-NPDR) in terms of mean blur rate (MBR) as determined by laser speckle flowgraphy (LSFG). The MBR of the optic nerve head (ONH) in the vessel and the tissue in the S-NPDR with panretinal photocoagulation (PRP) group was significantly lower than that in the S-NPDR without PRP and normal subjects groups (A) (B) (*p* < 0.001, *p* < 0.001, respectively). The choroidal MBR in the S-NPDR with PRP group was significantly lower than that in the S-NPDR without PRP and the normal subjects groups (*p* = 0.028, *p* < 0.001).

The ONH MBR in the vessel area was 29.1 ± 9.0 (arbitrary unit, AU) in the S-NPDR with PRP group and was significantly lower than that of the S-NPDR without PRP and the normal subjects groups, which was 40.7 ± 7.7 AU and 41.5 ± 8.3 AU, respectively (*p* < 0.001) (Fig 3). The ONH MBR in the tissue area was 9.0 ± 2.7 AU in the S-NPDR with PRP group and was significantly lower than that in the S-NPDR without PRP and the normal subjects groups, which was 10.8 ± 2.4 AU and 11.4 ± 2.8 AU, respectively (*p* < 0.001, *p* < 0.001). The choroidal MBR was 6.4 ± 3.7 AU in the S-NPDR with PRP group and was significantly lower than that in the S-NPDR without PRP and the normal subjects groups, which was 7.8 ± 2.0 AU and 9.2 ± 3.7 AU, respectively (*p* = 0.028, *p* < 0.001). There were no significant differences in the ONH and choroidal MBR between the S-NPDR without PRP and the normal subjects groups.

### Comparison of choroidal morphology between eyes with diabetic retinopathy and normal subjects

**Fig 4:**
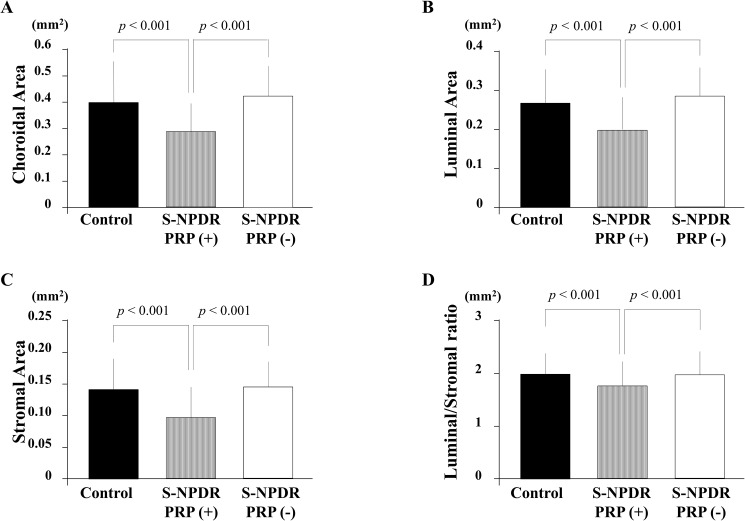
Differences between the eyes of normal subjects and those of patients with severe non-proliferative diabetic retinopathy (S-NPDR) with regard to subfoveal choroidal area as determined by binarization. The choroidal (A), luminal (B), and stromal area (C) in the S-NPDR with panretinal photocoagulation (PRP) group were significantly smaller than that in the S-NPDR without PRP and the normal subjects groups (*p* < 0.001, *p* < 0.001, *p* < 0.001, respectively). The luminal/stromal ratio in the S-NPDR with PRP group was significantly smaller than that in the S-NPDR without PRP and the normal subjects groups (*p* < 0.001, *p* < 0.001, respectively).

In the normal subjects group, the choroidal area, the luminal area, and the stromal area were 0.407 ± 0.145, 0.265 ± 0.098, and 0.138 ± 0.052 mm^2^, respectively (Fig 4). In the S-NPDR with PRP group, those were 0.289 ± 0.126, 0.189 ± 0.082, and 0.105 ± 0.046 mm^2^, respectively. In the S-NPDR without PRP group, those were 0.429 ± 0.087, 0.284 ± 0.061, and 0.145 ± 0.034 mm^2^, respectively. The areas determined by binarization in the S-NPDR with PRP group were significantly smaller than that in the S-NPDR without PRP and the normal subjects groups (*p* < 0.001, *p* < 0.001, *p* < 0.001, respectively). The luminal/stromal ratio in the S-NPDR with PRP group were significantly smaller than that in the S-NPDR without PRP and the normal subjects groups (*p* < 0.001, *p* < 0.001, respectively).

### Correlation of ocular blood flow with other parameters

The results of single linear regression analyses are displayed in the S-NPDR with PRP group in Tables 2 and 3. The ONH MBR in the vessel correlated with choroidal MBR (*r* = 0.295, *p* = 0.009), BCVA (*r* = −0.386, *p* < 0.001), number of PC shots (*r* = 0.345, *p* = 0.002), and duration of post-photocoagulation (*r* = −0.379, *p* < 0.001). The ONH MBR in the tissue correlated with choroidal MBR (*r* = 0.428, *p* < 0.001), gender (*r* = 0.256, *p* = 0.026), BCVA (*r* = −0.348, *p* < 0.001), luminal area (*r* = 0.243, *p* = 0.038), and duration of post-photocoagulation (*r* = −0.381, *p* < 0.001). The choroidal MBR correlated with BCVA (*r* = −0.339, *p* = 0.002).

**Table 2:** Result of Spearman’s rank correlation coefficient between the choroidal MBR and clinical parameters in patients with severe non-proliferative diabetic retinopathy.

Parameter		Choroid	Age	Gender	HbA1c	Duration	BCVA	AL	SFCT
		MBR				of DM			
**MBR**									
**ONH**	**vessel**	0.295^b^	-0.081	0.025	0.167	0.005	-0.386^a^	-0.119	0.177
	**tissue**	0.428^a^	-0.026	0.256^c^	0.151	0.009	-0.348^a^	-0.100	0.214
**Choroid**		-	-0.069	0.091	-0.068	-0.013	-0.339^b^	-0.006	0.067

MBR = mean blur rate; ONH = optic nerve head; DM = diabetes mellitus; BCVA = best corrected visual acuity; AL = axial length; SFCT = subfoveal choroidal thickness, ^a^*p* < 0.001, ^b^*p* <0.01, ^c^*p* < 0.05.

**Table 3:** Result of Spearman’s rank correlation coefficient between the choroidal MBR and clinical parameters in patients with severe non-proliferative diabetic retinopathy.

Parameter		IOP	Choroidal	Luminal	Stromal	MOPP	HR	Number of	Duration
			Area	Area	Area			PC shots	after PC
**MBR**									
**ONH**	**vessel**	-0.041	0.142	0.145	0.123	-0.025	-0.064	-0.345^b^	-0.379^a^
	**tissue**	0.060	0.227	0.243^c^	0.177	0.131	0.100	-0.195	-0.381^a^
**Choroid**		0.047	0.054	0.059	0.040	0.113	0.167	-0.027	-0.105

MBR = mean blur rate; ONH = optic nerve head; IOP = intraocular pressure; MOPP = mean ocular perfusion pressure; HR = heart rate; PC = photocoagulation, ^a^*p* < 0.001, ^b^*p* <0.01, ^c^*p* < 0.05.

**Fig 5:**
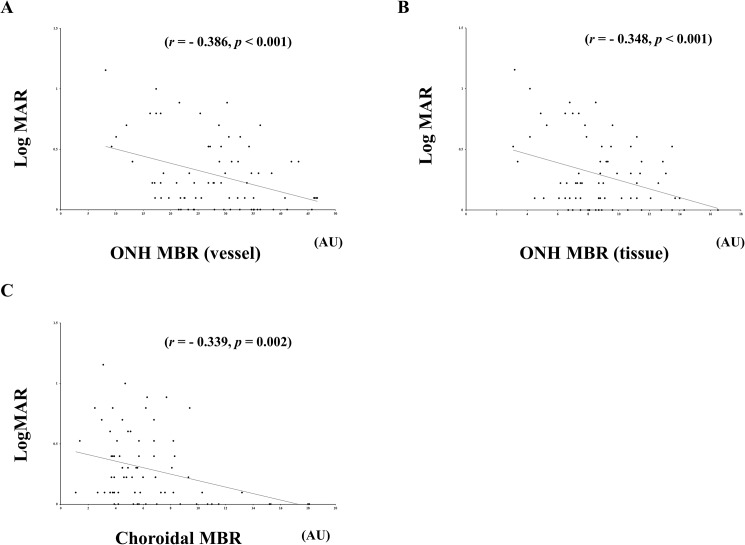
Relationship between best-corrected visual acuity (BCVA), optic nerve head (ONH), and choroid mean blur rate (MBR). The BCVA correlated with ONH MBR in the vessel (*r* = −0.386, *p* < 0.001), in the tissue (*r* = −0.348, *p* < 0.001), and choroidal MBR (*r* = −0.339, *p* = 0.002). AU = arbitrary units.

Fig 5 demonstrates the correlation between the ONH and choroidal MBR and LogMAR BCVA. The LogMAR BCVA had a significant negative correlation with ONH MBR in the vessel (*r* = −0.386, *p* < 0.001), in the tissue (*r* = −0.348, *p* < 0.001), and choroidal MBR (*r* = −0.339, *p* = 0.002).

Stepwise multiple regression analysis demonstrated that BCVA and the post-photocoagulation period were independent factors associated with ONH MBR in the vessel (Table 4)

**Table 4:** Results of multiple stepwise regression analysis for independence of factors contributing to ONH MBR.

Variable			
Dependent	Independent	*β*	*p—*value
ONH MBR (vessel)	Duration after PC	-0.406	< 0.001
	BCVA	-0.355	< 0.001
	Axial length	-0.137	0.184
	Age	0.131	0.208
	HbA1c	0.121	0.249
	Number of PC	0.128	0.288
	Stromal area	0.107	0.323
	Luminal area	0.098	0.353
	Heart rate	-0.067	0.514
	Gender	0.056	0.590
	SBP	0.028	0.784
	DBP	-0.020	0.844
	IOP	-0.020	0.872
	MOPP	0.010	0.920

ONH = optic nerve head; MBR = mean blur rate; PC = photocoagulation; BCVA = best corrected visual acuity; SBP = Systolic blood pressure; DBP = diastolic blood pressure; IOP = intraocular pressure; MOPP = mean ocular perfusion pressure.

BCVA, the post-photocoagulation period, and gender were independent factors associated with ONH MBR in the tissue (Table 5)

**Table 5:** Results of multiple stepwise regression analysis for independence of factors contributing to ONH MBR.

Variable			
Dependent	Independent	*β*	*p—*value
ONH MBR (tissue)	Duration after PC	-0.385	< 0.001
	BCVA	-0.327	0.001
	Gender	0.261	0.011
	SBP	0.174	0.086
	Age	0.136	0.198
	Axial length	-0.091	0.390
	Luminal area	0.065	0.560
	Stromal area	0.056	0.613
	DBP	-0.109	0.666
	MOPP	-0.135	0.666
	HbA1c	0.042	0.681
	IOP	0.007	0.715
	Heart rate	-0.026	0.809
	Number of PC	0.007	0.952

ONH = optic nerve head; MBR = mean blur rate; PC = photocoagulation; BCVA = best corrected visual acuity; SBP = Systolic blood pressure; DBP = diastolic blood pressure; MOPP = mean ocular perfusion pressure; IOP = intraocular pressure.

In addition, BCVA, number of photocoagulation, HR, and age were independent factors associated with choroidal MBR (Table 6).

**Table 6:** Results of multiple stepwise regression analysis for independence of factors contributing to choroidal MBR.

Variable			
Dependent	Independent	*β*	*p—*value
Choroidal MBR	BCVA	-0.537	< 0.001
	Number of PC	-0.344	0.008
	Heart rate	0.311	0.010
	Age	-0.264	0.029
	Duration after PC	—0.180	0.094
	HbA1c	-0.165	0.172
	Gender	0.138	0.203
	SBP	0.112	0.334
	MOPP	0.104	0.373
	DBP	0.107	0.382
	IOP	0.074	0.497
	Stromal area	0.073	0.513
	Luminal area	0.057	0.600
	Axial length	-0.042	0.719

MBR = mean blur rate; BCVA = best corrected visual acuity; PC = photocoagulation; SBP = Systolic blood pressure; MOPP = mean ocular perfusion pressure; IOP = intraocular pressure; DBP = diastolic blood pressure.

## Discussion

Our results showed that ONH and choroidal MBR were reduced in the PRP on eyes with S-NPDR but were not reduced in untreated eyes with S-NPDR. In addition, PRP on eyes with S-NPDR significantly reduced the SFCT and choroidal area as determined by binarization compared with untreated eyes with S-NPDR and normal eyes. Multiple stepwise regression analysis revealed that BCVA was a common independent factor associated with ONH and choroidal MBR in PRP on eyes with S-NPDR.

**Fig 6:**
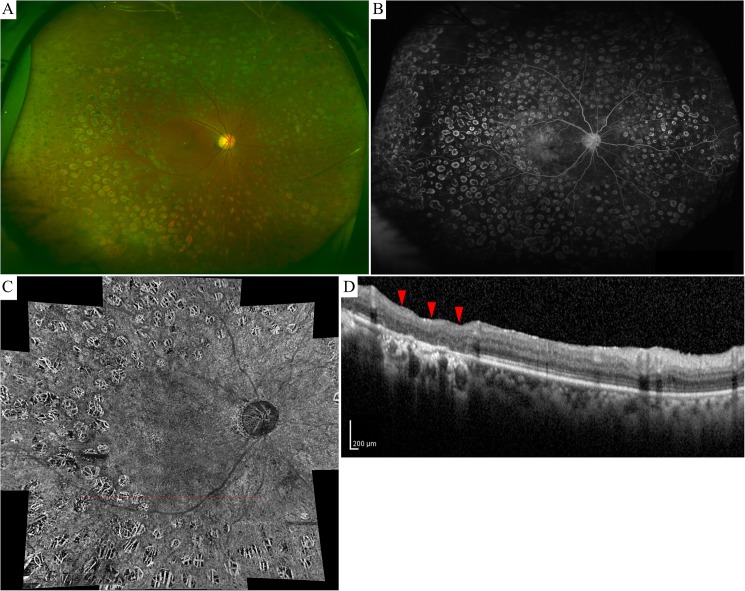
Optical coherence tomography angiography showing an eye with S-NPDR following PRP. Fundus photograph (A) and fluorescence angiography (B) taken with the Optomap® camera showing an eye with S-NPDR following PRP. Optical coherence tomography (OCT) angiography of the photocoagulated area showing choroidal major vessel because of the defect of RPE and choriocapillaris (C). An SD-OCT Spectralis® image was taken at the red line of the angiography (D). Photocoagulated regions (red arrow head) indicates absence of the outer nuclear layer and photoreceptor cells with the inner retinal layers lying in close apposition to Bruch's membrane and disruption of RPE layer and choriocapillaris.

It has been reported that photocoagulation essentially eliminated the choriocapillaris, when assessed by either laser scanning ophthalmoscopy indocyanine green angiography or counts of microspheres in the choroid.[34] Histologic damage to the choriocapillaris in humans have also been reported for such lesions.[35, 36] Morphological studies have reported that the outer nuclear layer, the high oxygen-consuming photoreceptor cells, and RPE were absent following PRP along with obliteration of the choriocapillaris,[35–37] suggesting that the ocular blood flow in the photocoagulated area decreases after PRP. Our OCT and OCT angiography map clearly demonstrates the long-term disruption of photoreceptor cells, RPE, and choriocapillaris in burn regions following PRP (Fig 6), corroborating these histologic findings.[37] Accordingly, these findings can be interpreted as a result of the large number of photocoagulation shots, which cause a wide, disrupted area in these tissues, resulting in reduced retinal and choroidal blood flow in the lesion.

ONH MBR was reduced on S-NPDR eyes with PRP but was not reduced in untreated eyes with S-NPDR, and single linear regression analysis showed that the ONH MBR exhibited a significant negative correlation with the number of PC shots in the present study. Grunwald et al [9, 10] and Patel et al [38] showed a decrease in retinal blood flow in diabetic patients following complete fundus PRP; some other experimental reports also corroborate these findings. [39, 40] Fujio et al [11] reported that regional laser treatment produces a regional reduction in retinal blood flow ranging from 60% to 78%, consistent with measurements of preretinal and intraretinal oxygen tension, which have indicated increases in oxygen over photocoagulated regions. These reports are in good agreement with our results.

Data on choroidal blood flow at the macula area following PRP varies among previous reports. Flower et al [41] reported that the effect of coagulating the peripheral retinal area markedly increased the choriocapillaris blood flow in the central area of the fundus relative to that in the periphery in monkey eyes as evidenced by indocyanine green angiography. Using a reflection spectra method, Augsten et al [42] also reported that peripheral retinal photocoagulation improved choroidal circulation in the macular area in patients with S-NPDR. Foveal choroidal blood flow measured using laser Doppler flowmetry was reported to increase one month following PRP. [43] On the other hand, it has been reported that the choroidal blood flow was significantly lower in PRP-treated eyes compared with that in untreated eyes as measured using laser interferometry and Color Doppler imaging. [44–46]

One possible explanation for this variety is that choroid blood flow at the macula area following PRP might be associated with PRP-induced inflammation and the measurement period. In most studies, blood flow was measured relatively early, such as 1 to 3 months following PRP, and it cannot be denied that the inflammation induced by photocoagulation had not resolved at the time of measuring. In addition, in the short term following PRP e.g. 1 week, it might be responsible for choroidal swelling, probably due to a shifting of blood vessels from the peripheral choroid to the foveal area. [47] Furthermore, the measuring methods, instruments, measured regions, and disease processes were different in each study, and this may contribute to the variability of the results. However, few reports are available describing post-PRP long-term choroidal blood flow at the macula area upon complete resolution of inflammation. The mean post-photocoagulation period is approximately 9 years in the present study, and the choroid blood flow at the macula was significantly decreased in S-NPDR patients, especially with impaired vision.

Earlier studies reported a significant thinner choroid following PRP on eyes with S-NPDR relative to untreated eyes with S-NPDR. [48–51] These results are in good agreement with our results, showing that PRP on eyes with S-NPDR significantly reduced SFCT and the stromal and luminal areas as determined by binarization, and compared with that in untreated eyes with S-NPDR and normal eyes. It has been reported that choroidal blood flow determined by LSFG significantly positively correlated with SFCT in a larger number of normal eyes. [52] We did not compare choroidal blood flow before and after PRP: however, there was no difference in choroidal blood flow between untreated eyes with S-NPDR and normal eyes. Accordingly, it is most likely that PRP treatment is related with the significantly reduced choroidal blood flow and decreased SFCT in PRP-treated S-NPDR eyes.

The present study demonstrated that the ONH MBR and the choroidal MBR significantly correlated with BCVA in PRP on eyes with S-NPDR patients, and BCVA in PRP-treated eyes with S-NPDR was worse than that in untreated eyes with S-NPDR. There are several possible explanations for this. First, the retina and ONH in PRP-treated eyes with S-NPDR with good vision would have smaller areas of non-perfusion prior to PRP and would need only little photocoagulation, with a relatively high blood flow remaining. Second, the ocular blood flow in S-NPDR patient eyes with impaired vision would already have been reduced prior to PRP. Third, eyes which require a larger number of photocoagulation would be predisposed to cause macular edema and the disruption of outer retinal layer e.g. ellipsoid zone, resulting in lower vision, because BCVA in untreated eyes with S-NPDR did not decrease compared with normal eyes, implying that PRP itself may decrease vision. Fourth, a high ocular blood flow is needed to maintain a good vision. Oxygen required by photoreceptors in the fovea is supplied from the choroid. Ooto et al [53] used an adaptive optics scanning laser ophthalmoscope to determine cone photoreceptor density, compared their findings with microstructures determined by a commercially available SD-OCT, and suggested that cone density in the foveal area correlates with BCVA. A large number of photoreceptors would require a higher choroidal blood flow for photoreceptor survival.

The post-photocoagulation period negatively correlated with ONH MBR in our multiple regression analysis. The photocoagulation effect was not limited to the lesioned area, but extended outside of the area by at least 1 to 2 mm.[34] Reportedly, 70% of laser scars increase in size on serial examinations performed over periods ranging from 2 to 81 months.[54] With photocoagulation for diabetic retinopathy, there is usually some damage to the underlying choroid and temporary closure of the choriocapillaris on the irradiated area. If the damage is sufficient to destroy a lobule in the choriocapillaris, the RPE overlying this lobule adjacent to the treatment site could later on become atrophic and contribute to this RPE atrophic creep. [54] The expanding atrophic creep would be related to decreasing retinal blood flow. On the other hand, there was no correlation between the post-photocoagulation period and the choroidal MBR. Although the reason is unclear, we evaluated only macular choroidal blood flow; changes in choroidal blood flow in other areas remain unknown, which might result in no correlation between the factors.

There are several limitations to this study. First, our study is cross-sectional, i.e., parameters may vary among the individual. Accordingly, the investigation of the relationship between changes in ocular blood flow with the post-photocoagulation period requires longitudinal study data. Second, we used a rectangle at the fovea to measure the MBR of choroidal blood flow, and the area binarized included 1,500 μm surrounding the fovea, meaning that choroid measurements were performed only in the center, not in the lesioned area or in the entire choroid. Third, we did not evaluate the situation before and after PRP with regard to VEGF concentration in the vitreous. Therefore, the relationship between the BCVA and VEGF remains unclear. Further longitudinal studies using a larger number of subjects will be necessary for clarification.

In conclusion, the ONH and choroidal MBR in addition to choroidal component, including the luminal area, was significantly lower in eyes of patients with S-NPDR after PRP compared with normal subjects. This could suggest that the significantly reduced ocular blood flow in PRP-treated S-NPDR eyes correlated with long-term decreased post-PRP luminal area and visual acuity.
